# Public health policy should be grounded in established evidence that acknowledges uncertainty: a response to The Lancet series on ultra-processed foods

**DOI:** 10.1186/s12916-026-05214-y

**Published:** 2026-09-28

**Authors:** Daniel B. Ibsen, Michael Fridén, Georgia D. Tomova, Natalia Ortega, Peter W.G. Tennant

**Affiliations:** 1 Department of Public Health, Aarhus University, Aarhus, Denmark; 2 Steno Diabetes Center Aarhus, Aarhus University Hospital, Aarhus, Denmark; 3 Department of Clinical Medicine, Aarhus University, Aarhus, Denmark; 4 Department of Public Health and Caring Sciences, Uppsala University, Uppsala, Sweden; 5 Centre for Longitudinal Studies, UCL Social Research Institute, University College London, London, UK; 6 Division of Preventive Medicine, Department of Medicine, Brigham and Women’s Hospital, Boston, MA USA; 7 Yale School of Public Health, Yale University, New Haven, CT USA; 8 Leeds Institute for Data Analytics, University of Leeds, Leeds, UK; 9 School of Medicine, University of Leeds, Leeds, UK

**Keywords:** Ultra-processed food, Nova, Nutritional epidemiology

## Abstract

**Background:**

The recent three-part Lancet series on ultra-processed foods (UPFs) argued that the displacement of traditional diets by UPFs is a major driver of poor diet quality and diet-related chronic diseases, which is becoming a major public health challenge worldwide. This Debate article aims to highlight key uncertainties surrounding that evidence base and the causal interpretation of the reported associations.

**Main body:**

We discuss uncertainties related to three core questions central for making causal claims: what are UPFs, what are the mechanisms and what is the right comparison group? We argue that the breadth of the Nova classification system as an exposure framework encompasses nutritionally and functionally diverse foods that are unlikely to share common biological effects. This heterogeneity complicates causal inferences and risks conflating well-established dietary risk factors with more speculative attributes related to processing, additives, and food structure. Many of the proposed mechanisms linking UPFs to adverse health outcomes are not unique to ultra-processing and may operate similarly across foods regardless of processing level. While much emphasis has been given to the comparison between minimally processed and UPF, the comparison between processed food and UPF may better isolate the effects of ultra-processing. By embracing these uncertainties, we outline a way forward focusing on disaggregation of UPFs into specific components and characteristics.

**Conclusions:**

Advancing the evidence base in this way will support clearer attribution of risk and more targeted interventions. Public health policy should remain grounded in well-characterised dietary exposures while transparently acknowledging uncertainty around ultra-processing as an independent causal factor.

## Background

In November 2025, *The Lancet* published a three-part series on ultra-processed foods (UPFs), their commercial determinants, and their links to human health [1]. In the first of these, Monteiro et al., hypothesise that UPFs are displacing ‘*long-established diets’*, leading to deteriorating diet quality, and higher risks of multiple diet-related chronic diseases [2]. Judging that the ‘*evidence fulfils seven of the nine Bradford Hill criteria to infer causality’,* they call for ‘*policies that promote and protect dietary patterns based on a variety of whole foods’* and urge that ‘*the need for further evidence should not delay public health action’*.

These are strong claims that underestimate the importance of grounding public health policy in established evidence that acknowledges uncertainty. While there is no doubt that diet plays a major role in human health and chronic disease, and that dietary interventions offer a powerful means to improve population health, the specific contribution of ultra-processing, defined by the Nova classification, as a cause and target for intervention is far less clear. Is an ultra-processed chocolate cake materially more harmful than a home-made one? Is minimally-processed salty meat preferable to ultra-processed multigrain bread? These inconvenient questions may be frustrating for those seeking urgent action to improve population health, but ignoring such nuance is not without risks; especially given the fragility of public trust in science. Nutritional epidemiology has a long history of confident but mistaken causal claims that demands caution [3]. Future efforts to improve population health through dietary intervention must therefore be grounded in an evidence-based approach, built on acknowledging what we do and do not know. Several recent papers have highlighted the limitations of the research on UPFs, but none has done so with a causal inference perspective [4–7]. In the following, we consider some of the key limitations for making causal claims glossed over by Monteiro et al. and discuss how these inform more practical options for future research and public health intervention.

## Main text

### What are UPFs?

The central challenge with studying and interpreting the causal effects of UPFs on health stems from the broad definition of the exposure provided by the Nova classification [8]. Monteiro et al. describe foods in Nova group 4 as comprising “*branded*,* commercial formulations made from cheap ingredients extracted or derived from whole foods and combined with additives*”.[2 ] While this may describe a commercially coherent cluster of foods, it is not biochemically coherent. UPFs hence incorporate a range of different foods and features, each of which may have different effects on human health. The consequence is that several foods that have long been implicated in adverse health outcomes, such as processed meat [9], sugar-sweetened beverages [10], and sugary foods [11], are combined alongside other foods with far less evidence of harm, such as dairy products and bread [12, 13]. It also conflates known problems, such as high fat, salt, sugar, and calorific intake, with a range of speculative and less well understood concerns around food texture, processing, and additives. In theoretical terms, this means that UPFs are a multidimensional exposure that is arguably fundamentally unsuitable for causal enquiry [14]. In practical terms, it means that studies of UPFs produce averaged and context-specific results that may downplay the larger effects of certain harmful foods or features, while incriminating foods or features with neutral or even beneficial effects [14]. This makes it difficult to judge how past research translates to potential policies in different food environments, especially if certain foods and features are already subject to dietary guidelines and legislation.

### What are the mechanisms?

This fundamental challenge with UPFs begs the question, what specific mechanisms drive the association between UPFs and adverse health outcomes? Monteiro et al. argue such knowledge is not necessary for immediate public health action and invokes many historical examples where positive public health interventions have been introduced before firm mechanisms have been established [2]. But we already know that many Nova 4 foods are harmful for well-known reasons with clear biological mechanisms. The snag is that most of these mechanisms are not unique to UPFs. High energy density, for example, is one proposed way that UPFs are thought to increase the risk of future chronic disease, through increased energy intake and weight gain. But UPFs are not the only high energy density foods. A homemade chocolate cake can have equally high energy density as one that is store-bought and factory-manufactured, and may have an equal effect on weight gain. In a recent randomised crossover trial that compared two diets that both adhered to UK dietary guidelines - one consisting of mostly minimally processed food and one consisting of mostly UPFs - both groups experienced weight loss after 8 weeks, with the minimally processed food group experiencing 0.96 kg greater weight loss [15]. Soft food texture is also implicated in the association between UPFs and weight gain. Similarly, there are several examples of soft-textured minimally processed and Nova 3 processed foods, such as fruit smoothies or homemade bread. In fact, evidence from randomised trials suggests that food texture, not ultra-processing, could be one of the key drivers of higher energy intake [16]. In a recent controlled dietary intervention study, where UPFs and non-UPFs were matched on the degree of hyper-palatability, energy density, and texture, no difference in *ad libitum* energy intake was observed among US adolescents consuming a buffet meal [17]. 

### What is the right comparison group?

In response to such criticisms, Monteiro et al. propose the hypothesis that, while a Nova 4 food is not automatically worse than another with a lower Nova category, the Nova 4 version of a particular food will always be worse than the particular food’s lower Nova equivalent. Although this hypothesis is appealing, it is not, however, currently supported by evidence. Relatively few studies compare individual UPF foods with direct non-UPF equivalents. Most of those that do are trials, and not all comparisons demonstrate that UPF foods are necessarily worse than similar but less processed alternatives. Comparisons of plant-based meat with unprocessed red meat [18], plant-based milk with cow’s milk [19], and artificially sweetened beverages with water [20], have not observed any short-term differences in health outcomes. This points to a deeper issue with the evidence base; most studies of the health implications of UPFs compare Nova 4 foods to Nova 1 foods, but it may be unrealistic to expect people to substitute easily available shop-bought food with home-made meals from minimally processed ingredients if their livelihood does not support this and any isolated policy that assumes they will is unlikely to succeed. If ultra-processing, and the use of industrial additives, are truly the cause of adverse health, then this should be detectable by comparing outcomes between Nova 4 and similar Nova 3 foods. Such research would also be much more directly relevant for population health, offering simple paths to reformulation and dietary guidelines, rather than calling for populations to reverse the profound social and economic changes that underpin the changing food environments.

## Conclusions

The consumption of soft, hyperpalatable, energy-dense foods that are high in fat, sugar, and salt is almost certain to be harmful to health. Many foods that meet these criteria are UPFs, and for many people reducing UPF intake will therefore probably be beneficial, by reducing exposure to such foods. It is not clear, however, whether there are additional features of UPFs that make them uniquely harmful compared with less processed foods with otherwise similar properties.

To address this, we suggest that future research should move away from studying UPFs as a broad category and seek to examine specific subtypes and features, such as texture and additives (see Table 1). In recent work, Monteiro and colleagues explicitly argue against analyses of subtypes of UPFs, citing methodological challenges such as confounding and measurement bias [21]. But these challenges are not resolved by producing and studying monolithic variables such as the Nova classification. Indeed, the opposite may be true. It is far harder, for example, to eliminate confounding between groups with very different exposure profiles (e.g. highest versus lowest quartile of UPF intake) than between more modest contrasts (e.g. processed versus unprocessed red meat). Similarly, the impact of measurement bias can be exacerbated when creating a summary variable from a series of mismeasured components if the errors are correlated, as is typical in dietary data [22]. 

**Table 1:** Key uncertainties in UPF research and constructive ways forward

Uncertainty	Ways forward	Examples
What is the exposure?	Investigate health effects of specific Nova 4 foods and food components, rather than treating UPFs as a single coherent exposure.	High-protein yoghurt drink (Nova 4) versus plain Greek yoghurt (Nova 1); fortified soya milk (Nova 4) versus plain soya milk (Nova 1)
What is the right comparator?	Compare specific Nova 4 foods with their Nova 3 equivalents to test whether ultra-processing per se confers additional harm beyond known nutritional risk factors.	Wholegrain packaged bread (Nova 4) versus wholegrain artisan bread (Nova 3); flavoured yoghurt (Nova 4) versus plain yoghurt (Nova 3)
What causes the harm?	Conduct well-controlled feeding studies that isolate specific features of UPFs (such as texture, additive content, or energy density) to establish whether proposed mechanisms of harm are unique to ultra-processing or shared with less processed foods.	Soft-textured diet versus hard-textured diet matched for nutrients, additives, and energy density; factory-made jam with additives versus factory-made jam without additives

Where evidence is uncertain, as with UPFs, it is essential that scientists acknowledge that uncertainty and seek to address it. Indeed, evidence suggests that communicating uncertainty protects rather than undermines public trust in science, and that unwarranted certainty is what most generates scepticism [23]. While we agree that the food system must change towards healthier foods, we do not agree this is necessarily best served by a generic targeting of UPFs. There is a real risk that an emphasis on UPFs as a broad category distracts from existing dietary guidance and from considering targeted interventions with stronger evidential foundations. Taxes on sugar-sweetened beverages, salt reformulation targets, and front-of-pack labelling schemes demonstrate how acting on specific, well-characterised exposures can meaningfully improve population health [24]. These approaches do not require resolving the contested question of whether ultra-processing itself is harmful, and offer clearer and more actionable paths forward than asking people to personally navigate the uncertain boundaries of the Nova classification.

## Data Availability

No datasets were generated or analysed during the current study.

## Abbreviations

UPF: Ultra-processed food
